# Bioexploration and Phylogenetic Placement of Entomopathogenic Fungi of the Genus *Beauveria* in Soils of Lebanon Cedar Forests

**DOI:** 10.3390/jof7110924

**Published:** 2021-10-31

**Authors:** Charbel Al Khoury, Georges Nemer, Richard Humber, Nehme El-Hachem, Jacques Guillot, Racha Chehab, Elise Noujeim, Yara El Khoury, Wadih Skaff, Nathalie Estephan, Nabil Nemer

**Affiliations:** 1 Department of Natural Sciences, Byblos Campus, School of Arts and Sciences, Lebanese American University, Byblos P.O. Box 36, Lebanon; 2 Division of Genomics and Translational Biomedicine, College of Health and Life Sciences, Hamad Bin Khalifa University, Doha P.O. Box 34110, Qatar; GNemer@hbku.edu.qa; 3 Department of Biochemistry and Molecular Genetics, American University of Beirut, Beirut P.O. Box 110236, Lebanon; hachemn@gmail.com; 4 Robert W. Holley Center for Agriculture and Health, USDA-ARS Emerging Pests and Pathogens Research Unit, 538 Tower Road, Ithaca, NY 14853-2901, USA; rah3@cornell.edu; 5 Department of Dermatology Parasitology and Mycology, Oniris, Ecole Nationale Vétérinaire, Agroalimentaire et de l’Alimentation, P.O. Box 44307 Nantes, France; jacques.guillot@oniris-nantes.fr; 6 Department of Agriculture and Food Engineering, Holy Spirit University of Kaslik, Jounieh P.O. Box 446, Lebanon; racha_chehab@hotmail.com; 7 National Center for Marine Sciences, National Council for Scientific Research—CNRS, Beirut P.O. Box 11-8281, Lebanon; enjeim@cnrs.edu.lb (E.N.); khouryaragro@gmail.com (Y.E.K.); 8 Dipartimento di Scienze del Suolo, della Pianta e degli Alimenti (Di.S.S.P.A.), Università degli Studi di Bari “Aldo Moro”, P.O. Box 70126 Bari, Italy; 9 Ecole Supérieure d’Ingénieurs d’Agronomie Méditerranéenne, Université Saint Joseph, Taanayel, Zahlé P.O. Box 159, Lebanon; wadih.skaff@usj.edu.lb; 10 Department of Chemistry and Biochemistry, Faculty of Arts and Sciences, Holy Spirit University of Kaslik, Jounieh P.O. Box 446, Lebanon; nathalieestephan@usek.edu.lb

**Keywords:** soil-bioexploration, phylogeny, new-taxa, *Beauveria tannourinensis*, *Beauveria ehdenensis*

## Abstract

The cedar forests of Lebanon have been threatened by the outbreak caused by climate change of a web-spinning sawfly, *Cephalcia tannourinensis* (Hymenoptera: Pamphiliidae), which negatively impacted the survival of one of the oldest tree species on earth. In this study, we investigated the occurrence of naturally soil-inhabiting entomopathogenic fungi for their role in containing the massive outbreak of this insect. We used a combination of fungal bioexploration methods, including insect bait and selective media. Morphological features and multilocus phylogeny—based on Sanger sequencing of the transcripts encoding the translation elongation factor 1-alpha (*TEF-α*), RNA polymerase II second largest subunit (*RBP2*), and the nuclear intergenic region (Bloc) were used for species identification. The occurrence rate of entomopathogenic fungi (EPF) varied with location, soil structure, forest structure, and isolation method. From 15 soil samples positive for fungal occurrence, a total of 249 isolates was obtained from all locations using different isolation methods. The phylogenetic analysis confirmed the existence of two novel indigenous species: *Beauveria tannourinensis* sp. nov. and *Beauveria ehdenensis* sp. nov. In conclusion, the present survey was successful (1) in optimizing the isolation methods for EPF, (2) investigating the natural occurrence of *Beauveria* spp. in outbreak areas of *C. tannourinensis*, and (3) in characterizing the presence of new *Beauveria* species in Lebanese cedar forest soil.

## 1. Introduction

Soil, the living sphere of microorganisms, provides shelter for a diverse array of bacteria, actinomycetes, fungi, algae, and protozoa [1] including species of the cosmopolitan entomopathogenic fungal genus *Beauveria* (Hypocreales: Cordycipitaceae) [2]. *Beauveria bassiana*—the type species of this genus—has been widely studied as a bio-control agent, and currently forms the basis of several commercially available mycoinsecticides [3]. The wide range of hosts susceptible to *Beauveria* includes over 15 insect [4] and mites [5] orders. Furthermore, *Beauveria* produces a wide range of mycotoxins, including, but not limited to, beauvericin and bassianolide, that are involved in key developmental stages of the fungus and that have demonstrated pharmacological and therapeutic potentials [6,7,8].

Rehner et al. [2] identified phylogenetic relationships within morphologically classified species of *Beauveria* based on the multisequence of RNA polymerase II largest subunit (*RBP1*), RNA polymerase II second largest subunit (*RBP2*), translation elongation factor alpha (*TEF-α*), and Bloc nuclear intergenic region. Including a few new taxa described since that phylogenetically based revision of the genus, 14 well-supported terminal lineages are now formally recognized: *B. amorpha, B. bassiana, B. brongniartii, B. caledonica, B. malawiensis, B. vermiconia, B. asiatica, B. australis, B. kipukae, B. pseudobassiana, B. sungii*, *B. varroae*, *B. lii*, and *B. hoplocheli* [2,9,10].

In Lebanon, there were no studies on the taxonomy of entomopathogenic fungi (EPF) until the identification of LTB01, an entomopathogenic strain of *Beauveria* infecting larvae of cedar sawfly, *Cephalcia tannourinensis* Chevin (Hymenoptera: Pamphiliidae), from a Tannourine Cedar Forest Nature Reserve [11]. During the last three decades and until now, the endemic *C. tannourinensis*, the first *Cephalcia* species reported on *Cedrus libani* (Pinales: Pinaceae), has been considered the principal defoliator insect of the emblematic cedars in Lebanon. The infestation affects about 70% of the forest, with the major concern of its rapid spread and the subsequent infestation of the remaining cedar forests within the Mediterranean region [12]. The most prevalent natural pathogen of *C. tannourinensis* is *Beauveria*, which infects all developmental stages of this pest [11]. During its life cycle, *Cephalcia* passes most of its time as prepupae in diapause inside an earth-walled chamber in the mineral layer of the soil and, hence, is directly affected by soil entomopathogens [13]. Even though *Beauveria* is the first and only identified natural enemy of *C. tannourinensis*, there is still considerable ambiguity with regard to the occurrence, as well as the phylogeny, of this natural pest regulator. The objectives of this study were to collect and to characterize *Beauveria* isolates from the soil of three Lebanese forests where outbreaks of *C. tannourinensis* caused substantial damage to the cedars.

## 2. Materials and Methods

### 2.1. Study Sites and Collection of Soil Samples

Three Lebanese forests were chosen for the collection of soil samples: the Tannourine Cedar Forest Nature Reserve (1600–1800 masl, precipitation: 850 mm; average temperature per year: 14 °C; area: 625 hectares), the Horch Ehden natural reserve (1400–1600 masl; precipitation: 1095 mm; average temperature per year: 18.6 °C; area: 200 hectares), and the Bcharre cedar forest (1950 masl; precipitation: 950 mm; average temperature per year: 13 °C; area: 11 hectares). The average age of cedar trees in the three forests is over 300 years with an exception to the old cedar forest in Bcharre where a few specimens are more than 1000 years old. Different plots were selected for each forest based on their soil structure and texture, forest structure, and surface of the natural reserve. Three plots were selected from Tannourine Cedar Forest Nature Reserve and were numbered T1 (N 34°12′, 517; E 35°55′, 699), T2 (N 34°12′, 485′; E 35°55′, 510), and T3 (N 34°12′, 311; E 35°56′, 125). Ehden Cedar Forest Nature Reserve was divided into two plots E1 (N 34°18′, 480; E 35°59′, 510) and E2 (N 34°18′, 550; E 35°59′, 721)), and Bcharre cedar forest was considered as one plot B1 (N 34°14′, 571; E 36°02′, 905). For each plot, 10 random collection sites were selected for soil sampling. Therefore, a total of 60 soil samples were collected from the six different plots (T1, T2, T3, E1, E2, and B1). This collection was repeated twice (two biological replicates), during May 2017 and May 2018, and resulted in a total of 120 soil samples. Soil weighing approximately 1 kg was collected for each sample from a depth of up to 15 cm using small shovels. Tools were disinfected with 70% ethanol between each sample collected, in order to avoid cross-contamination. The collected samples were placed in plastic bags and carried to the entomology laboratory of the Faculty of Agricultural and Food Sciences at Holy Spirit University of Kaslik for further treatment. Gravel, stones, and plant debris were eliminated by passing the soil through a 2 mm pore sieve. Sieves were sterilized with 70% ethanol and 1% sodium hypochlorite for 10 s and 3 min respectively, then rinsed twice with sterile distilled water between soil samples. Sieved soil samples were placed separately in new plastic bags, sealed, and stored at 4 °C until their analysis within no longer than 6 months.

### 2.2. Isolation of EPF from Soil by Means of Insect Bait

EPF were isolated from soil samples using the third instar larvae stages of *Galleria mellonella* [14] (Lepidoptera: Pyralidae), prepared as described by Meyling and Eilenberg [15], and naturally collected prenymphal stages of *C. tannourinensis* as baits. Soil samples were moistened with sterile distilled water. From each soil sample, sub-samples of 60 g each were deposited into new sterile plastic containers. Two repetitions (two plastic containers) were made for each bait method. The top 2 cm of each plastic container were intentionally left empty to allow for proper ventilation. Two individuals of each bait kind (two third instar larval stages of *G. mellonella* or two prenymphal stages of *C. tannourinensis)* were placed on the soil in each plastic container, making it a total of four insects for each baiting method utilized per soil sample. The plastic containers were closed and incubated in dark conditions for fungal growth (25 °C, 90% RH), and inverted daily to maximize the interaction between spores and insects. Infection of insects was recorded for 3 weeks. This test was repeated in two biological replicates (two different soil collections from May 2017 and May 2018), making it a total of 8 insects for each baiting method utilized. Dead insects were isolated from containers, placed in 70% ethanol and 1% sodium hypochlorite for 10 s and 15 s respectively, then rinsed twice with sterile distilled water. Each disinfected larva was placed in a sterile Petri dish containing filter paper Whatman No. 1 and incubated at 25 °C with 90% RH. Once sporulation occurred on the infected subjects, a sample of conidia was inoculated onto potato dextrose agar (PDA) medium culture using a sterile loop. Potato dextrose agar cultures were incubated at 25 °C, and this was considered as an isolate. Discs (6 mm) from the vigorously growing cultures of nonsporulating *Beauveria* cultures were placed in sterile cryotubes containing 10% glycerol in water and maintained at −80 °C at the Plant Pathology Fungal Collection of the Post Herbarium at the American University of Beirut.

### 2.3. Isolation of EPF from Soil by Means of Selective Media

Various media were prepared for isolation of EPF directly from soil: (i) dodine-based medium originally described by Beilharzet al. [16] to isolate *Beauveria* spp. and confirmed by several studies [17,18,19,20]; (ii) CTAB-based medium [21]; and (iii) Low sugar-content medium (DOC2) [22]. For soil homogenization, 10 g of soil were placed into 90 mL of sterile water and shaken at 150 rpm for 15 min to release fungal propagules from the soil matrix. Using a sterile pipette, 1 mL of aliquots were removed and spread on the three different selective media using a sterile loop. Cultures were incubated in total darkness to promote fungal growth (25 °C, RH 90%) for 7 days. Three repetitions were made for each aliquot sample (three Petri dishes of each selective medium). This test was repeated in two biological replicates (two different soil collections from May 2017 and May 2018), making it a total of six Petri dishes of each medium.

### 2.4. Morphological Identification of Fungal Isolates

The morphological examination of each isolate was determined based on macro- and microscopic identification criteria [23]. Mycelium was removed from an in vitro culture using a sterile loop, placed onto a glass slide in a drop of lactophenol and covered with a coverslip. Microscopic measurements of conidiogenous cells and conidia were taken from the agar cultures at 15 days post-inoculation.

Conidia were suspended in 0.01% Tween and mixed with an equal volume of molten (70 °C) 0.1% Nusieve GTG agarose. A transparent adhesive tape preparation stained with lactophenol was performed for the observation of fungal cells. Thirty conidia from 15-day old cultures were measured for each sample. This experiment was done in triplicates, making it a total of 90 conidia measured for each isolate.

### 2.5. Molecular Characterization

For the phylogenetic analysis, a subsample of isolates was used. Fifteen soil samples (out of 60) were positive for *Beauveria* spp. Only one isolate from each of the 15 positive soil samples was randomly selected for further analysis. Potato dextrose broth (PDB) was used to grow isolates for DNA extraction. Erlenmeyer flasks containing 25 mL of PDB were inoculated with 0.5 cm plugs of fungus from 15-day-old cultures described above and incubated at 25 °C for 7 days to promote fungal growth. The mycelium was harvested by centrifugation of the medium on 8000 rpm for 10 min. To remove any residual medium, the mycelium was washed twice with sterile distilled water. The mycelium was frozen at −20 °C until further use. DNA extraction was performed using DNeasy kit (QIAGEN, GmbH, Hilden, Germany) following the manufacturer’s instructions. Sequences of two protein-coding genes (*RBP2* and *TEF-α*) were used because of their demonstrated utility in phylogenetic placement of *Beauveria* spp [24], and the nuclear intergenic region Bloc for cryptic speciation review within *B. bassiana* [2,25]. For all loci, PCR amplifications were done in a total volume of 20 µL, which included 10 µL PCR Master Mix 2x Thermo Scientific, MA, USA, Taq DNA polymerase (0.05 U/µL), reaction buffer, 4 mM MgCl_2_, 0.4 mM of each dNTP, nuclease-free water), 20 ng of the genomic DNA, 10 pmol each of the opposing amplification primers (Table 1), and 7 µL of distilled water. Amplification cycles, using CFX96 Real-Time System (BIO-RAD, CA, USA), were performed according to Rehner et al. [2]. All PCR reaction volumes were separated on a 1.5% agarose gel to check the success of amplification reactions. All PCR products were purified using the QIAquick PCR Purification kit (QIAGEN, GmbH, Hilden, Germany) following the manufacturer’s instructions. The purified amplicons were quantified, and Sanger sequencing with either the forward or reverse primers were undertaken at the molecular core facility at the American University of Beirut using ABI3500 instrument as previously described [26].

### 2.6. Phylogenetic Analysis

Phylogenetic analyses were performed on sequences representing 14 species of *Beauveria* and the outgroup taxon *Cordyceps cicadae*. The three-locus datasets (Bloc, *TEF-α*, *RPB2*) DNA sequences were edited and aligned with Muscle version 3.2 [30] using MEGA version 7 (Molecular Evolutionary Genetic Analysis) [31]. All three sequences were concatenated using sequenceMatrix [32], and the resulting concatenated sequences with 5814 nucleotide sites were explored in the subsequent phylogenetic analysis (TreeBASE ID: 24955). Maximum-likelihood (ML) trees were computed with IQTREE (version 1.6.12), which relies on novel and improved algorithms to estimate maximum likelihood phylogenies. Briefly, it takes a multiple sequence alignment as input, and the appropriate model of DNA substitution was obtained with ModelFinder [33] from the Bayesian Information Criterion (BIC). The TPM2u+F+I+G4 was selected as best fitting model for the concatenated dataset. Finally, the ML tree was constructed from the best fitting model and analyses were conducted with 1000 bootstrap replicates [34]. Maximum parsimony (MP) and non-parametric bootstrapping (MP BS) (1000 replicates), to assess clade support, were executed with PAUP* 4.0a [35]. The gaps were excluded, and all nucleotides were unordered and equally weighted. All MP searches were performed using 1000 random taxon-addition sequence replicates with tree-bisection and reconnection branch-swapping (TBR). To find out if data partition could be combined, a conditional combination criterion was utilized [36]. Data were combined if (i) monophyly statement were congruent or (ii) >70% bootstrapping score was recorded with one or both monophyletic groups. A Bayesian approach (MB) was also performed using MrBayes 3.2.7 [37] with two runs of Markov chains (diagnostic calculated every 200 generations) for 30 × 10^6^ generations (standard deviation < 0.01). The lambda exponential rate parameter for the branch-length prior was calculated as described by Brown et al. [38]. Traces were examined graphically with Tracer 1.6 [39], and trees generated before stabilization of ML Scores were discarded. The paired sites test, Shimodaira and Hasegawa test, was conducted using PAUP* 4.0a to compare topologies obtained with the three different statistical approaches used in this study [40]. The significant difference in the sum of site-wise log-likelihoods for all trees was evaluated by bootstrap sampling of site scores (RELL sampling) with 1000 replicates [41].

### 2.7. Statistical Analysis

Data relevant to the presence/absence of *Beauveria* using the different extraction methods was calculated as percentages in each forest. A chi-square of homogeneity test was applied to determine if the presence or absence of *Beauveria* differed between the various sampled forest habitats after adjusting the computing *p* values by the Bonferroni method and using the Z-test to compare the percentages. To check for the best selective method of extraction, a Chi-Square Goodness of fit was performed after computing the frequencies of *Beauveria* isolated by the four methods. Then, a multiple binomial test was executed after adjusting the computed *p*-values with the Bonferroni method to detect the significance between pairs of extraction methods. Data relevant to the species of *Beauveria* using the different extraction methods were analyzed by the statistical comparison test of means (ANOVA). The Tukey test was used at the 5% threshold for the separation of means.

## 3. Results

### 3.1. Occurrence of EPF

We used two insect baits and three selective media to isolate EPF from the soil in three different locations in Lebanon. The results obtained demonstrated the wide presence of EPF strains including *Beauveria* spp., *Cordyceps fumosorosea* (accession number *TEF-α*: MK975968) and saprophytic fungi such as *Fusarium* spp., *Aspergillus* spp., and *Pythium* spp. All saprophytic fungi were, however, considered as contaminants and discarded from future analysis; only *Beauveria* spp. were kept for analysis. The percentage of occurrence and number of detected species was significantly affected by the isolation method. (X^2^(3) = 12.031; *p* = 0.007) taking into account that the low sugar medium (DOC2) was not included in the analysis since no fungi was extracted by this medium. Overall, a total of 249 isolates was recorded from the six different locations. Based on the number of isolates, the occurrence of *Beauveria* isolates in sampling locations indicated that *Beauveria* was undetectable in plot T3 in Tannourine Cedar Forest Nature Reserve and Bcharre Cedar Forest regardless of the method of isolation used.

The isolation of *Beauveria* with the *G. mellonella* method, the *C. tannourinensis* method, the dodine medium method, as well as the CTAB medium method was highly statistically dependent on the site location (Tannourine, Ehden, and Bcharre) (Table 2). DOC2 isolation medium method was not successful in isolating *Beauveria* from any sites. The chi square test of homogeneity for Tannourine Cedar Forest indicated that the presence or absence of *Beauveria* is very highly significantly dependent on the considered plot in the forest for each of the selected isolation methods. Thus, each extraction method was affected by the habitat (plot) whereby plot 1 and plot 2 harbored more *Beauveria* than plot 3 irrespective of the extraction method used (*C. tannourinensis*: X^2^ = 19.46, df = 2, *p* < 0.05; *G. mellonella*: X^2^ = 26.97, df = 2, *p* < 0.05; dodine: X^2^ = 37.6, df = 2, *p* < 0.05; CTAB: X^2^ = 22.7, df = 2, *p* < 0.05). The same applies for the cedar forest of Ehden which also indicated that the presence or absence of the EPF is significantly dependent of the considered habitat (plot), for each of the selected isolation methods (*C. tannourinensis*: X^2^ = 3.93, df = 2, *p* < 0.05; *G. mellonella*: X^2^ = 9.26, df = 2, *p* < 0.05; dodine: X^2^ = 16.88, df = 2, *p* < 0.05; CTAB: X^2^ = 9.22, df = 2, *p* < 0.05).

To determine the best selective method of extraction in all cedar forests, results showed that there is a high statistically significant difference between levels of *Beauveria* extracted by the four methods (*C. tannourinensis*, *G. mellonella*, dodine, and CTAB) with a chi-square goodness of fit $\;\left(\chi^{2}\left(3\right)=12.031;p=0.007\right)$. There was a significant difference (p<0.008) between *Beauveria* frequencies detected by *C. tannourinensis* (lowest one) and dodine (highest one) extraction methods. No significant differences were detected between all the other combinations of methods of extraction.

**Figure 1:**
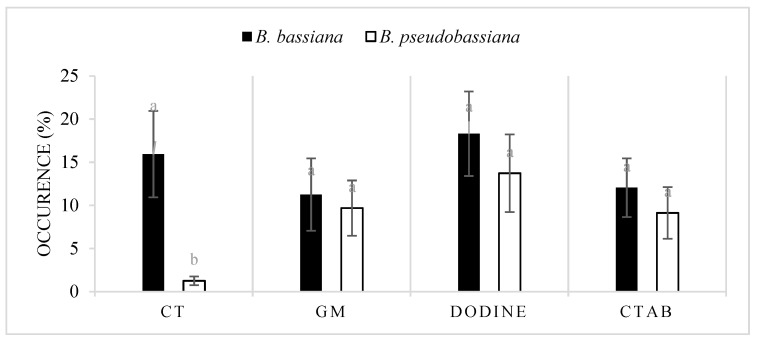
Occurrence rate of *B. bassiana* and *B. pseudobassiana* in the Lebanese soils using different isolation methods (CT: *Cephalcia tannourinensis* bait method, GM: *Galleria mellonella* bait method, dodine: dodine-based selective medium, and CTAB: CTAB-based selective medium). The values with different superscript letters in a column are significantly different (*p <* 0.05).

There was no significant difference between the occurrence of *B. bassiana* and *B. pseudobassiana* using *G. mellonella* (F = 0.85, df = 1, *p* > 0.05), dodine (F = 0.461, df = 1, *p* > 0.05), and CTAB (F = 0.405, df = 1, *p* > 0.05). This result was significant exclusively using the *C. tannourinensis* bait method for the isolation of *Beauveria* spp. (F = 8.013, df = 1, *p* < 0.05) (Figure 1).

### 3.2. Morphological Characterization of Beauveria Isolates

The morphological description of ex-type cultures has been previously reported by Rehner et al. [2]. Due to its structural simplicity and lack of phenotypical differences, *Beauveria* isolates cannot be identified to the species level by their morphologies; however, the conidial shape and size can be considered as key morphological features shared by a few of the species in this genus. *Beauveria bassiana* conidia averaged 1.98 × 1.33 µm (1.82–2.23 × 0.9–1.56 µm), mostly ellipsoidal and rarely forming spherical spore clusters among aerial hyphae, and colorless in mass. *Beauveria pseudobassiana* conidia averaged 2.00 × 1.90 µm (1.93–2.21 × 1.85–2.00 µm) with mostly globose spore clusters among aerial hyphae, and colorless in mass.

### 3.3. Phylogenetic Placement of Isolates

In order to further characterize the isolates, we carried Sanger sequencing of the *TEF-α, RBP2*, Bloc, and Internal transcribed spacer “rDNA ITS1-5.8S-ITS2 (ITS barcode)” loci. The sequences were uploaded to the NCBI portal (Table 3), and phylogenetic analyses were undertaken to place them on an evolutionary tree. The *ITS* sequences were not included in the phylogenetic analysis because of their limited informative value within hypocrealean ingroups [2].

**Figure 2:**
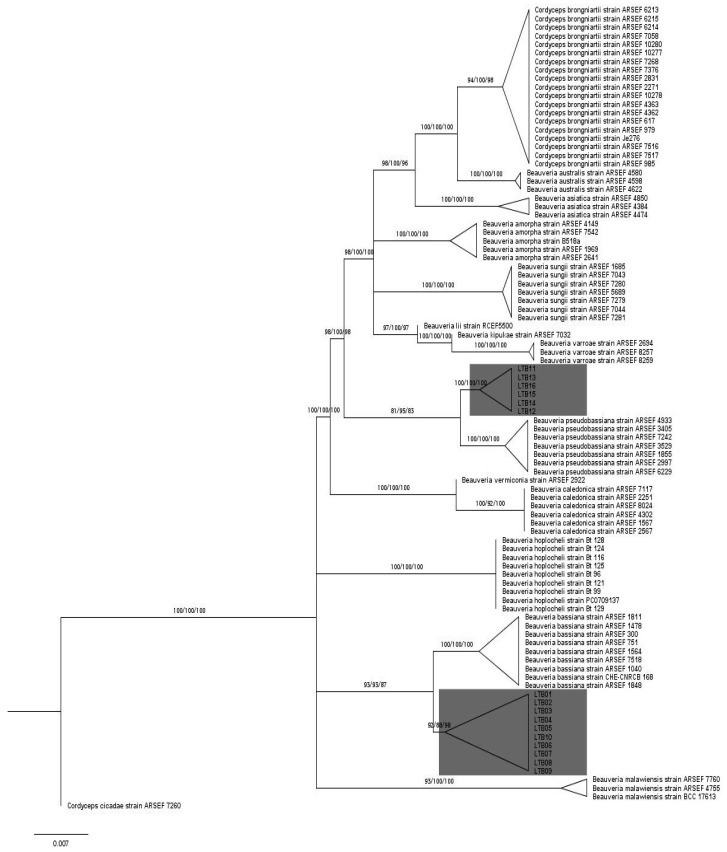
Phylogenetic tree of the *Beauveria* species showing the relationships between isolates from Lebanon and species described by Rehner et al. [2], Zhang et al. [9], and Robène-Soustrade et al. [10], in addition to an outgroup taxon: *Cordyceps cicadea*. The tree was inferred with the maximum likelihood method (TPM2u+F+I+G4 model) from the concatenated dataset including, *TEF-α*, *RPB2*, and Bloc sequences. Maximum likelihood (ML), maximum parsimony (MP), and Bayesian approach (MB) produced congruent trees (*p* < 0.05). The maximum likelihood (ML) method produced the corresponding cladogram from the concatenated sequences and visualized with tree application. Values next to branches indicate ML bootstrap percentage, MP bootstrap percentage, and MB posterior probabilities, respectively. Triangles represent the collapsed clades.

The SH test showed that all approaches utilized in this study produced congruent trees (*p* < 0.05). The phylogenetic tree, built upon the ML approach, and generated from the concatenated dataset clustered *Beauveria* species into 16 distinct and robust groups independent of approach (ML, MP, and MB), supported by ML and MP bootstrap percentages in addition to MB posterior probabilities. Fourteen out of 16 groups corresponded to the *Beauveria* spp. previously described [2,9,10]. The two remaining groups corresponded to the two distinct indigenous groups comprising 10 (LTB01, LTB02, LTB03, LTB04, LTB05, LTB06, LTB07, LTB08, LTB09, LTB10) and 6 (LTB11, LTB12, LTB13, LTB14, LTB15, LTB16) isolates, clustered distinctly from the other *Beauveria* groups, although these two groups were related to *B. bassiana* and *B. pseudobassiana*, respectively, according to the ML tree (Figure 2).

The branching patterns 93/93/87 and 81/95/83 within *Beauveria* LTB01 to LTB10 and LTB11 to LTB16, respectively, are highly supportive of new lineages. These branches were assigned with ML bootstrap percentage, MP bootstrap percentage and, MB posterior probabilities

Morphological features coupled to a detailed molecular phylogenetic analysis allowed us to describe novel indigenous species *Beauveria.* These species were classified as new species referred to herein as *B. tannourinensis* (LTB01 to LTB10) and *B. ehdenensis* (LTB11 to LTB16).

### 3.4. Taxonomy

*Beauveria tannourinensis* Al Khoury C, Nemer G, Humber R, Guillot J, Nemer N, sp. nov.

#### 3.4.1. Mycobank: MB 832258

Holotype: BT0517 Lebanon: Batroun, North Lebanon (34°12′46.3″ N 35°55′44.3″ E), on a *C. tannourinensis* nymph (Hymenoptera: Pamphiliidae) in Tannourine Cedar Forest Nature Reserve, 14 May 2017.

Ex-type culture LTB02, deposited in the Plant Pathology Fungal Collection of the Post Herbarium at the American University of Beirut. Sequences from LTB02 strain were deposited in GenBank under accession numbers: *RPB2* = MK908082, Bloc = MK884864, ITS = MK884879, and *TEF-α* = MK975955.

Colony characteristics on full strength PDA, 36–42 mm diam at 10 d at 23 °C, appearance densely cushion-like and up to 7 mm thick, and uncolored. The reverse is white at first, then becoming yellow at margins and brown at the center. Odor indistinct. Conidia 1.98 × 1.33 (2.23–1.82 × 1.56–0.9) µm, aggregated in mostly ellipsoidal and rarely spherical clusters among aerial hyphae and white in mass. Vegetative hyphae septate, branched, hyaline, smooth-walled, 1–2 µm wide. Conidiogenous cells are solitary in but usually in dense clusters of five or more, base subspherical to ampulliform and 3–5 µm wide, apex with an indeterminate 1 µm wide geniculate, denticulate rachis, produced laterally on aerial hyphae or from subtending cells.

*Beauveria ehdenensis* Al Khoury C., Nemer G, Humber R., Guillot J, Nemer N, sp. nov.

#### 3.4.2. Mycobank: MB 832259

Holotype: BE0518 Lebanon.: Zgharta, North Lebanon (34°18′42.1″ N, 35°59′03.3″ E), on a *G. mellonella* third instar larva (Lepidoptera: Pyralidae) in Horsh Ehden Nature Reserve, 10 May 2018.

Ex-type culture LTB15, deposited in the Plant Pathology Fungal Collection of the Post Herbarium at the American University of Beirut. Sequences from LTB15 strain were deposited in GenBank under accession numbers: *RPB2* = MK908085, Bloc = MK884867, ITS = MK884883, and *TEF-α* = MK975958.

Colony characteristics on full strength PDA, 48–53 mm diam at 10 d at 23 °C, appearance densely dotted-like and up to 2 mm thick, and uncolored. The reverse is white at the center and margins. Odor indistinct. Conidia 2 × 1.9 µm (1.93–2.21 × 1.85–2) µm, aggregated in mostly globose clusters among aerial hyphae and white in mass. Vegetative hyphae septate, branched, hyaline, smooth-walled, 1–2 µm wide. Conidiogenous cells solitary in but usually in dense clusters of five or more, base subspherical to ampulliform and 3–4.5 µm wide, apex with an indeterminate 1 µm wide geniculate, denticulate rachis, produced laterally on aerial hyphae or from subtending cells.

## 4. Discussion

### 4.1. Bioexploration of EPF

The present study reports the results of the first bioexploration of entomopathogenic *Beauveria* species in Lebanon. A total of 249 isolates were recovered from soils of three Lebanese cedar forests using different isolation methods. Similar occurrences have been previously reported by Keller et al. [17], Rath et al. [42], Bidochka and Kasperski [43], Pérez-González et al. [44], Niemczyk et al. [45], and Sánchez-Peña et al. [46]. *Beauveria* isolates were recovered from 25% of the soil samples. In addition, the present study has identified that *Beauveria* strains isolated from the cedar forests are quite different from described species on the morphological and genetical levels. An in-depth study of fungal–insect relationships in the cedars’ ecological natural vegetation might enhance our understanding of the evolution and diversity of these fungi.

The evaluation of the selective medium for culturing *Beauveria* spp. showed that the dodine-based medium is the most efficient among the three media tested. This result is in accordance with the studies from Keller et al. [17] Enkerli et al. [20]. Strasser et al. [47] showed that dodine-based media can be used successfully to isolate *Beauveria* spp. and *Metarhizium* spp. from soil and insect cadavers. Moreover, a low level of contamination by saprophytic fungi has been also recorded using this medium. Isolation of EPF was also successful using a CTAB-based medium. This result is in accordance with the study from Posadas et al. [21]. However, CTAB-based medium was less selective because it allowed the development of many contaminant fungi. Shimazu and Sato [22] developed a DOC2 medium based on low sugar content and high pH supplemented with CuCl_2_ and crystal violet for the selective isolation of *Beauveria* spp. This medium has been successfully used by Safavi [48] to recover a new isolate of *Beauveria bassiana*. Surprisingly, in the present study, the DOC2 medium did not allow the growth of any single colony of *Beauveria* spp. Local conditions might have affected the tolerance ability of indigenous strains, inhibiting the possibility of their development on such an unconventional medium. In addition, dodine is not readily available in many countries in contrast to the ingredients for DOC2 medium, whose ingredients are widely available. Therefore, advanced studies should be held, trying slight modifications to the DOC2 medium, making it suitable to the development of indigenous EPF.

Two insect bait methods were used in the present study. The first one included highly susceptible larvae of the wax moth, *G. mellonella* [15,17,49]. This traditional method was able to recover *Beauveria* spp. from different Lebanese soils, and *B. bassiana* and *B. pseudobassiana* were both recovered at similar rates. The present study also included the first use of web-spinning sawfly, *C. tannourinensis* that proved to be a highly efficient bait method in isolating *Beauveria* from the Tannourine Cedar Forest. After the molecular analysis, most of the *Beauveria* isolated by the *Cephalcia* bait method turned out to be closely related to *B. bassiana* whereas the *Galleria* bait method showed an equal isolation between the species closely related to *B. bassiana* and *B. pseudobassiana*. This could be due to the fact that *C. tannourinensis* is more vulnerable to *B. bassiana* than to *B. pseudobassiana*. This is in accordance with findings reported by Al Khoury et al. [50] who showed that local isolates of *Beauveria* are relatively active against the eggs of *S. scabiei*.

### 4.2. Identification and Phylogenetic Placement

Macroscopic and microscopic examination, as well as multilocus phylogenetic analysis, proved that all *Beauveria* isolates from the Lebanese forests were distinct from previously described *Beauveria* species. In spite of morphological differences when collected for the first time from Tannourine Cedar Forest Nature Reserve in Lebanon, analyses of partial sequences of *ITS* and *TEF-α* loci identified LTB01 strain as *B. bassiana* Clade C [11,51]. Morphological characteristics of conidia were compared to ex-type cultures previously described by Rehner et al. [2], Zhang et al. [9], Robène-Soustrade et al. [10], Rehner and Buckley [24], and Rehner et al. [25] to confirm species assignments. The isolate LTB02 (representing the first lineage of isolates) produced mostly ellipsoidal conidia. Similar morphology was previously observed [11]. The ellipsoidal conidial shape has been previously reported for *B. brongniartii* (ARSEF 1431), *B. bassiana* (ARSEF 678), *B. asiatica* (ARSEF 4384), and *B. sungii* (ARSEF 1685) but the conidia produced by the indigenous Lebanese isolates were smaller. Furthermore, based on concatenated data of *TEF-α*, *RPB2*, and Bloc, *B. brongniartii, B. bassiana*, *B. asiatica*, and *B. sungii* represented four well supported terminal lineages, as described by Robène-Soustrade et al. [10] and Rehner et al. [2] and therefore were clearly separated from our local isolates. LTB15 isolate (representing the second lineage of isolates) produced mostly globose conidia with an average size of 2 × 1.9 µm. Although *B. pseudobassiana* is known to produce spherical conidia with an average size 2–3 × 1.5–2.5 µm, Robène-Soustrade et al. [10] reported *B. pseudobassiana* strain producing globose conidia. However, the latter strain (ARSEF 152) produced conidia with a larger size (3.5 × 3 µm) when compared to the conidia produced by the indigenous isolate (strain LTB15). Moreover, a study conducted by Al Khoury et al. [52] showed a significant difference in the expression of genes involved in the virulence of the local strain (LTB02) when compared to a commercially available strain of *B. bassiana.* This is also in good agreement with results obtained by Al Khoury et al. [53] demonstrating a significantly higher efficacy of the local strain against all developmental stages of *Tetranychus urticae* Koch (Acari: Trombidiformes) than to the commercial one (Tracer, Krishi Rasayan, India). These differences in the genetic expression, as well as bioactivities, could be considered as additional support that the local isolates of *Beauveria* are separated from previously described strains.

### 4.3. Occurence of EPF

A high incidence of EPF was recorded in the soil of the Tannourine Cedar Forest Nature Reserve. This forest may offer better environmental conditions for the development of EPF and/or have appropriate hosts for entomopathogenic fungal development exemplified by the massive presence of the diapausing stages of *C. tannourinensis* present in the soil. A higher incidence of EPF was recorded in the first and second plots (T1 and T2) than in the third plot (T3). A study conducted by Nemer et al. [12] showed that the soil samples from the T1 and T2 plots were of a clayey type and can be further classified into Red Mediterranean soil. It is possible that the soil’s light sandy structure in T3 is not suitable for the development of either EPF or for *C. tannourinensis*. This finding is consistent with previous findings showing that larvae of *C. tannourinensis* cannot survive in sandy soils [12]. Furthermore, these results are in accordance with the study from Strasser et al. [54] and Niemczyk et al. [45] suggesting that heavy clay soils could enhance the occurrence of EPF. Quesada-Moraga et al. [55], and Abdullah et al. [56] demonstrated that soils with higher organic matter content were favored by entomopathogenic fungal populations. A significant difference of fungal occurrence was observed between the two plots at Horch Ehden Natural Reserve (Table 2). Despite having the same environmental conditions, the occurrence of *Beauveria* spp. was higher in E1 where the habitat is composed mainly of cedars. Therefore, the high density of insect hosts in which the fungi can multiply has favored its occurrence at the location where Cedar trees and their pest are available. Even though these results differ from those reported by Bueno-Pallero [57] who hypothesized that vegetation type has no effect on EPF occurrence, they are consistent with those from Carrillo-Benitez et al. [58] and Pérez-Gonzalez et al. [44] who suggested that different *Beauveria* species may infect different insect hosts and adapt to specific pests and environments.

Several studies previously reported possible factors affecting the occurrence of *Beauveria* within different areas. Vega et al. [59] suggested that differences in environmental factors were the main reason for this fungal variation. Quesada-Moraga et al. [55] demonstrated the significant effect of soil properties on the isolation rate of EPF. In the present study, we demonstrated that the occurrence of EPF could also be affected significantly by using different isolation methods. These results are in line with those obtained by Bueno-Pallero et al. [57] who demonstrated that the utilization of selective media results in higher EPF occurrence rates relative to the baiting method. Our tests revealed that the best mass extraction method for *Beauveria* from the soil is the use of the dodine medium; however, for a species-specific isolation, baiting with a specific insect host would be recommended.

Since no bioexploration has been undertaken for EPF in Lebanon, this study could be considered as key for future bioexploration to elucidate the behavior of EPF in soil habitats. During the last three decades, in northern Lebanon, where more than 70% of the cedar forests occur these trees have been under threats from the defoliating insect, *C. tannourinensis* [13,60,61]. The only available control method is the use of endemic entomopathogens and in order to be able to integrate their usage within the cedars forest ecosystems, this study demonstrated that the presence of *Beauveria* species in the soil depends on complex interactions between biotic, abiotic, and edaphic factors. In their investigations into the utilization of *Beauveria* inoculum to control pest outbreak, Tartanus et al. [62] showed that the metabolic activity/versatility of fungi may also play a fundamental role in the soil abundance.

In conclusion, multiple factors affected the occurrence of EPF in this study beginning with the soil habitats, to the forest structure, the hosts, and the isolation methods. Combined results from morphological characteristics and multigene phylogenetic analysis successfully recovered two novel lineages that have been described here as the new species *Beauveria tannourinensis* and *Beauveria ehdenensis*. Much work remains to be conducted to study the behavior, survival, growth, and sporulation of these new entomopathogenic *Beauveria* species. Because the pest *C. tannourinensis* is being monitored regularly, a close monitoring of these entomopathogens is important in order to predict future outbreaks in these threatened cedar forest ecosystems.

## Figures and Tables

**Table 1:** Primers for PCR amplification and sequencing.

Locus	Primer	PCR	Sequence (5–3′) ^a^	Reference
Bloc	B5.1F	x	CGACCCGGCCAACTACTTTGA	[25]
	B3.1R	x	GTCTTCCAGTACCACTACGCC	[25]
	B822Ldg		AGATYCGYAACGTCAACTTT	[25]
	B22Udg		GTCGBAGCCAGAGCAACT	[25]
	B5.4F		CATTCGMGGCYTGTTCTTTGG	[25]
	BRn2		CTCCACGCATTCCGCACCAG	[25]
	Bfint		GTTCCTTGCCCTCGGTAATGAA	[25]
	BFn2		TCTCGATGCCGTTACCTACA	[25]
	Brint		AGCATATCGGGCATGACTGA	[25]
	BFn6		TGGTGCGGAATGCGTGGAGC	[25]
	B3.3R		TTCCAGTACCACTACGCCGGC	[25]
*Rbp2*	fRPB2_5F	x	GACGAYAGAGAYCAYTTYGG	[27]
	RPB2A_7cR	x	CCCATRGCTTGYTTRCCCAT	[27]
	fRPB2-7cF	x	ATGGGYAARCAAGCYATGGG	[27]
	RPB2-3053bR	x	TGRATYTTRTCRTCSACCAT	[28]
	Bv-RPB2A_R1		CCCCTGTTGATCATRAAGTCA	[25]
	Bv-RPB2A_F3		CCMGCCGARCCRCTYATTGA	[25]
	Bv-RPB2A_F4		CGCCTGAAGACDAARACMAACC	[25]
	Bv-RPB2B_R4		CRGCGTTRACAGRCACRATGA	[25]
	Bv-RPB2B_F1		AAGCGTCTTGATTTRGCRGGYCC	[25]
	Bv-RPB2B_R2		GCGTGAATYTTRTCRTCCAC	[25]
*TEF–α*	983F	x	GCYCCYGGHCAYCGTGAYTTYAT	[24]
	2218R	x	ATGACACCRACRGCRACRGTYTG	[24]
	1567RintB		ACHGTRCCRATACCACCRAT	[24]
	1577F		CARGAYGTBTACAAGATYGGTGG	[24]
ITS	ITS4	x	TCCTCCGCTTATTGATATGC	[29]
	ITS5	x	GGAAGTAAAAGTCGTAACAAGG	[29]

^a^ IUPCA degenerate nucleotides: D, AGT; H, ACT; N, ACGT; S, CG; R, AG; Y, CT.

**Table 2:** Occurrence frequency of *Beauveria* EPF recovered from soil samples.

Tannourine Cedar Forest Nature Reserve							
Plot	Soil Texture	Crop	Occurrence Frequency (%)				
			CT	GM	Dodine	Ctab	DOC2
T1	Clayey	Cedars	26.7 ^a^ *	31.7 ^a^	45 ^a^	28.3 ^a^	0 ^b^
T2	Clayey	Cedars	26.7 ^a^	36.7 ^a^	43.3 ^a^	31.7 ^a^	0 ^b^
T3	Sandy	Cedars	0 ^b^	0 ^b^	0 ^b^	0 ^b^	0 ^b^
**Horch Ehden Natural Reserve**							
**Plot**	**Soil Texture**	**Crop**	**Occurrence Frequency (%)**				
			**CT**	**GM**	**dodine**	**Ctab**	**DOC2**
E1	Clayey	Cedars	13.3 ^a^	18.2 ^a^	35 ^a^	21.7 ^a^	0
E2	Clayey	Pine	3.3 ^b^	1.7 ^b^	5 ^b^	3.3 ^b^	0
**Bcharre Cedar Forest**							
**Plot**	**Soil Texture**	**Crop**	**Occurrence Frequency (%)**				
			**CT**	**GM**	**Dodine**	**Ctab**	**DOC2**
B1	Clayey	Cedars	0	0	0	0	0

(CT: *Cephalcia tannourinensis* bait method; GM: *Galleria mellonella* bait method; dodine: dodine-based selective medium; and CTAB: CTAB-based selective medium). * Each subscript letter denotes a subset of habitat categories whose line proportions do not differ significantly from each other at the 0.05 level.

**Table 3:** Collection site and accession numbers (TEF-α, RBP2, Bloc, and ITS genes) of the representative strains.

Isolate	Area	Accession Number			
		TEF-α	RBP2	Bloc	ITS
LTB01	T1	EU177813	MK908095	MK884877	DQ984676
LTB02	T1	MK975955	MK908082	MK884864	MK884879
LTB03	T1	MK975954	MK908081	MK884863	MK884880
LTB04	T1	MK975964	MK908091	MK884873	MK884884
LTB05	T2	MK975960	MK908087	MK884869	MK884892
LTB06	T2	MK975965	MK908092	MK884874	MK884887
LTB07	T2	MK975959	MK908086	MK884868	MK884886
LTB08	E1	MK975961	MK908088	MK884870	MK884882
LTB09	E1	MK975962	MK908089	MK884871	MK884885
LTB10	E2	MK975963	MK908090	MK884872	MK884891
LTB11	T1	MK975956	MK908083	MK884865	MK884889
LTB12	T1	MK975953	MK908080	MK884862	MK884878
LTB13	T2	MK975966	MK908093	MK884875	MK884888
LTB14	T2	MK975967	MK908094	MK884876	MK884881
LTB15	E1	MK975958	MK908085	MK884867	MK884883
LTB16	E1	MK975957	MK908084	MK884866	MK884890
